# Heterogeneous Renal Trajectories in Pediatric IgA Nephropathy: A Single-Center Experience Highlighting the Dynamic Nature of Early Disease

**DOI:** 10.3390/pediatric18040084

**Published:** 2026-06-23

**Authors:** John Dotis, Antonia Kondou, Vasiliki Karava, Maria Tsirevelou, Ioannis Koutras, Olympia Dadoudi, George Liapis, Despoina Tramma, Maria Stamou, Nikoleta Printza

**Affiliations:** 1 Third Department of Pediatrics, Hippokration Hospital, Aristotle University of Thessaloniki, 54642 Thessaloniki, Greece; yandot@auth.gr; 2 First Department of Pediatrics, Hippokration Hospital, Aristotle University of Thessaloniki, 54642 Thessaloniki, Greece; antkontou@auth.gr (A.K.); mariatsir96@gmail.com (M.T.); 3 First Department of Pediatrics, “Aghia Sophia” Children’s Hospital, National and Kapodistrian University of Athens, 11527 Athens, Greece; vasilikikarava@hotmail.fr; 4 Radiology Department, Hippokration Hospital, 54642 Thessaloniki, Greece; gianniskoutras@yahoo.gr; 5 School of Medicine, University College of Dublin, Belfield Dublin, D04V1W8 Dublin, Ireland; olinadadoudi@gmail.com; 6 First Department of Pathology, Laikon Hospital, National and Kapodistrian University of Athens, 11567 Athens, Greece; pathology@laiko.gr; 7 Fourth Department of Pediatrics, Papageorgiou General Hospital, Aristotle University of Thessaloniki, 56429 Thessaloniki, Greece; dtramma@auth.gr; 8 Second Department of Pediatrics, AHEPA General Hospital, Aristotle University of Thessaloniki, 54636 Thessaloniki, Greece; bpedclin@auth.gr

**Keywords:** IgA nephropathy, pediatric nephrology, MEST-C classification, renal function, proteinuria, kidney biopsy, disease progression, chronic kidney disease

## Abstract

Background/Objectives: Pediatric IgA nephropathy (IgAN) is often considered to have a favorable early course. However, its progression is variable, and the prognostic value of histopathological classifications, such as MEST-C, remains incompletely defined in children. This study aimed to characterize clinicopathological features and the early disease course in pediatric IgAN and to descriptively examine histopathological findings and clinical outcomes. Methods: This retrospective, single-center study included children with biopsy-confirmed IgAN diagnosed between 2016 and 2025. Clinical, laboratory, and histopathological data were collected, and biopsies were assessed using the Oxford MEST-C classification. Follow-up data, including estimated glomerular filtration rate (eGFR), were analyzed descriptively, with follow-up extending from diagnosis to early 2026. Results: Fourteen patients were included, showing heterogeneous clinical presentations. Mesangial hypercellularity was observed in all cases (100%), with frequent endocapillary hypercellularity (78.6%) and segmental sclerosis (57.1%), consistent with a predominance of active lesions. Over a median follow-up of approximately five years, renal function remained stable in 57.1% of patients, declined in 21.4%, and improved in 14.3%, indicating variability in renal function during follow-up and potential reversibility in a subset of patients. One patient (7.1%) developed severe acute kidney injury requiring temporary dialysis, followed by full recovery. Given the descriptive design and limited sample size, no conclusions regarding associations between histopathological findings and renal outcomes can be drawn. Conclusions: Within this small cohort, pediatric IgAN showed variable renal function courses ranging from stability to decline or partial recovery. These findings should be considered descriptive and hypothesis-generating, supporting longitudinal monitoring in larger pediatric cohorts.

## 1. Introduction

Immunoglobulin A nephropathy (IgAN) is the most common primary glomerulonephritis worldwide and a major cause of chronic kidney disease (CKD) across all age groups [1,2]. It is characterized by mesangial deposition of IgA-containing immune complexes, triggering inflammatory responses that may lead to progressive glomerular injury [1]. Although it may occur at any age, IgAN is frequently diagnosed in children and adolescents, in whom clinical presentation, histopathological features, and disease course differ from those of adults [2,3].

In pediatric populations, IgAN typically presents with hematuria, often associated with intercurrent infections, while kidney function is usually preserved at diagnosis [4]. Children often exhibit active inflammatory lesions on biopsy, such as endocapillary hypercellularity and less chronic damage, including tubular atrophy and interstitial fibrosis [2,5]. Despite a generally favorable short-term course, IgAN in childhood cannot be considered uniformly benign. Long-term studies show that a substantial proportion of patients progress to CKD or kidney failure, particularly in the presence of persistent proteinuria or adverse histological features [1,3,4].

Kidney biopsy remains the gold standard for diagnosis and prognostic evaluation of IgAN [1]. The Oxford classification, updated to include crescents, is the standard histopathological scoring system, incorporating mesangial hypercellularity (M), endocapillary hypercellularity (E), segmental glomerulosclerosis (S), tubular atrophy and interstitial fibrosis (T), and crescents (C) (MEST-C) [6]. This classification has been evaluated across diverse populations and is integrated into current risk stratification approaches [7]. However, its prognostic value in pediatric IgAN remains incompletely defined, partly due to the relatively favorable early disease course and the influence of immunosuppressive therapies commonly used in children [4].

Studies examining clinicopathological correlations in IgAN have identified associations between specific histological lesions, particularly segmental sclerosis as well as tubulointerstitial damage, and adverse renal outcomes [3,7]. Nevertheless, most evidence derives from heterogeneous or predominantly adult cohorts, while pediatric data remain limited and sometimes inconsistent. In addition, variability in biopsy practices, treatment strategies, and follow-up duration complicates the interpretation and generalizability of prognostic markers in children [1]. These limitations highlight the need for well-characterized single-center cohorts with uniform histopathological assessment and detailed clinical follow-up.

In our previously published single-center experience of over 200 pediatric kidney biopsies, IgAN accounted for a consistent proportion (~10%) of biopsy-proven renal diagnoses [8]. Importantly, that study focused on the biopsy spectrum and did not include disease-specific clinicopathological correlations or standardized application of the MEST-C classification. The present study builds on this work by examining children with biopsy-proven IgAN over an extended period, uniformly classified according to the MEST-C system.

The aim of this study was to characterize the clinicopathological profile and early-to-middle-term course of pediatric IgA nephropathy in a single-center cohort, with emphasis on the description of Oxford MEST-C histological features and clinical outcomes. Patients were followed longitudinally from diagnosis to last available follow-up, capturing the full disease trajectory over a median period of approximately 5 years, with particular emphasis on persistent proteinuria during follow-up.

## 2. Materials and Methods

### 2.1. Study Design and Population

This investigation was designed as a retrospective observational study conducted at a single tertiary pediatric nephrology center. The study included pediatric patients diagnosed with IgAN confirmed by kidney biopsy over a ten-year period, from January 2016 through December 2025, with follow-up extending to early 2026. As a referral center for pediatric kidney disorders, the institution manages a broad spectrum of renal conditions in children using established clinical protocols, and the study population reflects a referral-center cohort with a relatively high burden of disease severity. Although the overall diagnostic and management approach remained consistent over the study period, minor adaptations in clinical practice and treatment protocols may have occurred over time, reflecting evolving evidence and guideline updates. These changes were not systematically evaluated and may have contributed to variability in individual patient outcomes. Therefore, they should be considered a potential source of heterogeneity when interpreting longitudinal renal trajectories and clinical outcomes.

Patients were eligible if they were younger than 18 years at the time of diagnosis and had complete baseline clinical, laboratory, and histopathological information, along with available follow-up data. Individuals with IgA deposition secondary to systemic conditions, including hepatic disease or systemic autoimmune diseases associated with secondary IgA nephropathy, were excluded. Patients with IgA vasculitis, including those with IgA vasculitis nephritis, were also explicitly excluded to ensure a homogeneous cohort of primary IgAN. Cases were also omitted if biopsy samples were inadequate for proper histological evaluation. Consequently, the study population represents a consecutive series of children with biopsy-confirmed primary IgAΝ treated at this center.

### 2.2. Data Collection and Clinical Variables

Clinical information was systematically collected from both electronic databases and paper-based medical records using a predefined data extraction framework. Baseline characteristics included demographic data (age and sex) and clinical presentation at diagnosis. Presenting features were categorized as isolated hematuria, isolated proteinuria, or the coexistence of both.

Additional clinical parameters recorded at baseline included blood pressure measurements and any history of infections preceding disease onset. Laboratory data at diagnosis encompassed serum creatinine levels, estimation of glomerular filtration rate (eGFR), and quantification of urinary protein excretion. Kidney function was calculated using the bedside Schwartz equation.

Longitudinal follow-up data were obtained for all patients, including repeated assessments of renal function, trends in proteinuria, and therapeutic interventions. The study period spanned from the date of initial diagnosis (starting in January 2016) to the latest available clinical follow-up in early 2026. The duration of follow-up varied among participants based on the timing of diagnosis, ranging from 1 to 10 years, with a median follow-up of approximately five years. Individual renal trajectories were evaluated using all available follow-up data for each patient, irrespective of follow-up duration. This approach enabled a patient-level descriptive assessment of disease course.

### 2.3. Histopathological Assessment

All participants underwent renal biopsy as part of routine diagnostic evaluation. Tissue samples were analyzed using light microscopy, immunofluorescence techniques, and electron microscopy in accordance with standard laboratory procedures.

Histological findings were categorized based on the Oxford Classification system, incorporating the MEST-C scoring framework. This system evaluates mesangial hypercellularity (M), endocapillary proliferation (E), segmental sclerosis (S), tubular atrophy/interstitial fibrosis (T), and the presence of crescents (C). Each parameter was graded according to established criteria (M0/M1, E0/E1, S0/S1, T0–T2, and C0–C2).

All biopsy specimens were reviewed by experienced renal pathologists as part of routine clinical assessment. To ensure clinical relevance, particular attention was given to the coexistence of active inflammatory lesions (E1, C) and chronic structural lesions (S1, T) as part of the histopathological characterization of the cohort. The resulting histopathological data were recorded systematically to characterize disease severity.

### 2.4. Definitions and Outcomes

Renal function was assessed using eGFR derived from serum creatinine, calculated with age-appropriate equations. Reduced kidney function was defined as eGFR < 90 mL/min/1.73 m^2^. Persistent proteinuria was defined as sustained protein excretion during follow-up despite therapeutic intervention. Hypertension was identified using standardized pediatric reference values adjusted for age, sex, and height. Episodes of acute kidney injury (AKI) were determined based on documented clinical events and significant transient changes in renal function. A key outcome of interest was partial or complete recovery of renal function, defined as a sustained improvement in eGFR following initial presentation or complete recovery after an AKI event.

The primary aim of this study was descriptive, focusing on the clinical and histopathological features of pediatric IgAΝ and their progression over time. Given the phenotypic diversity of the disease, the analysis focused on describing renal function during follow-up rather than identifying independent prognostic factors, providing a descriptive patient-level characterization of outcomes despite the limited cohort size. For descriptive purposes, renal outcomes were categorized according to the percentage change in eGFR during follow-up (<10% decline, 10–25% decline, and >25% decline). These categories were selected pragmatically to facilitate descriptive assessment of changes in kidney function over time and do not represent validated prognostic thresholds in pediatric IgAN.

### 2.5. Treatment

Information regarding treatment strategies was collected for all patients. This included the use of corticosteroids, adjunctive immunosuppressive medications such as mycophenolate mofetil (MMF), and therapies targeting the renin–angiotensin system. Renin–angiotensin system blockade with angiotensin-converting enzyme inhibitors (ACE inhibitors) was administered either for the management of arterial hypertension or for antiproteinuric purposes in normotensive patients, reflecting individualized therapeutic indications. ACE inhibitors were used during follow-up in accordance with clinical indications, including persistent proteinuria and/or hypertension.

Management decisions were made at the discretion of the clinical team in accordance with routine practice, with treatment tailored to individual patient characteristics and disease severity. Given the retrospective nature of the study, no standardized treatment protocol was imposed. ACE inhibitors were generally not initiated during periods of acute deterioration in renal function and were introduced or reintroduced after stabilization to reduce the risk of AKI. Treatment exposure was recorded descriptively without formal assessment of treatment effects. Adjustments in therapy over time, including dose reduction, discontinuation of corticosteroids, and continuation of immunosuppressive agents, were documented for descriptive purposes. Because treatment strategies were individualized and no untreated comparison group was available, the study was not designed to distinguish the natural history of the disease from potential treatment-related effects on renal outcomes.

### 2.6. Statistical Analysis

Data were summarized using descriptive statistical methods. Change in eGFR (ΔeGFR, %) was calculated as the percentage change between baseline and last follow-up values. Continuous variables are presented as either means with standard deviations or medians with interquartile ranges, depending on data distribution. Categorical variables are expressed as counts and percentages.

Given the limited number of cases (*n* = 14) and the significant phenotypic diversity of the cohort, formal inferential statistical testing and multivariable analyses were intentionally avoided to prevent overinterpretation of underpowered data. Instead, the analysis focused on a descriptive characterization of clinical, histopathological, and longitudinal features. Associations between clinical and histopathological features were explored descriptively. Formal correlation analyses (Pearson or Spearman) were not performed because of the limited sample size and the low frequency of several histopathological categories, which would have resulted in unstable and potentially misleading estimates. All statistical analyses were performed using IBM SPSS Statistics for Windows, version 28.0 (IBM Corp., Armonk, NY, USA).

### 2.7. Ethical Considerations

The study adhered to the ethical principles outlined in the Declaration of Helsinki. Owing to the retrospective nature of the study and the use of fully anonymized patient data, formal ethics committee review and approval were not required in accordance with institutional policies. The requirement for informed consent was waived in accordance with institutional guidelines. All data were handled with strict confidentiality, and no information that could identify individual patients was included in the analysis.

## 3. Results

A total of 14 pediatric patients with biopsy-proven IgAN were included, with diagnoses established between 2016 and 2025 (Table 1). The cohort exhibited marked variability in clinical presentation, ranging from isolated urinary abnormalities to nephrotic-range proteinuria and episodes of acute kidney injury. Macroscopic hematuria was the predominant presenting feature, frequently associated with infection-related triggers, while nephrotic-range proteinuria was observed in a considerable proportion of patients.

Histological evaluation revealed M1 in all cases (100%), with E1 present in 78.6% (*n* = 11) and S1 in 57.1% (*n* = 8), indicating a predominance of active histopathological lesions (Table 2).

**Figure 1:**
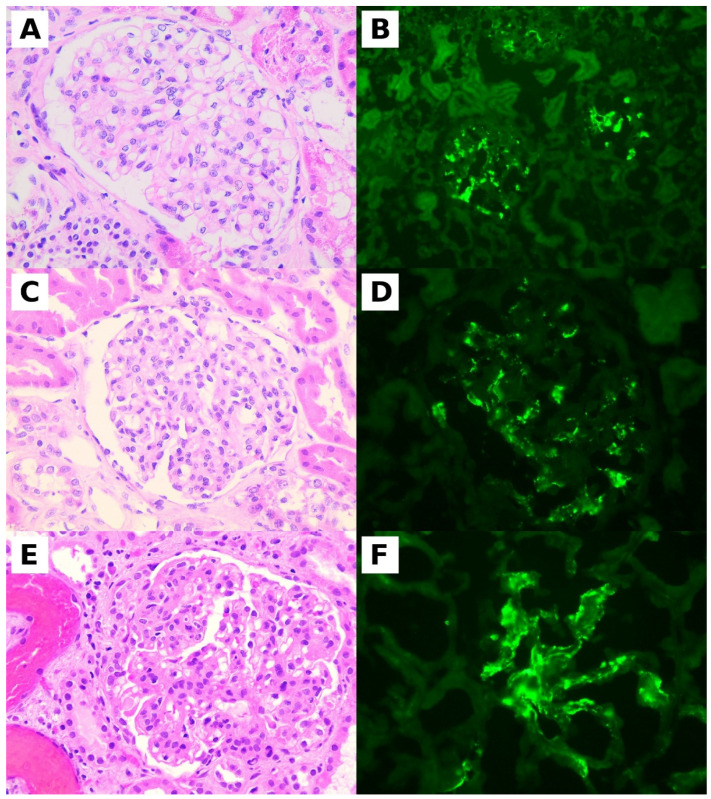
Representative histopathological findings in pediatric IgA nephropathy illustrating different Oxford MEST-C patterns. (**A**,**B**) Kidney biopsy from a patient with M1E0S0T0C0 lesions: (**A**) Light microscopy (hematoxylin and eosin stain, ×400) demonstrating mesangial hypercellularity. (**B**) Immunofluorescence microscopy showing mesangial IgA deposition using a fluorescein isothiocyanate (FITC)-conjugated anti-IgA antibody (×200). (**C**,**D**) Kidney biopsy from a patient with M1E1S1T0C0 lesions: (**C**) Light microscopy (hematoxylin and eosin stain, ×400) demonstrating mesangial and endocapillary hypercellularity. (**D**) Immunofluorescence microscopy showing prominent mesangial IgA deposition using a FITC-conjugated anti-IgA antibody (×400). (**E**,**F**) Kidney biopsy from a patient with M1E1S1T1C2 lesions: (**E**) Light microscopy (hematoxylin and eosin stain, ×400) demonstrating marked mesangial and endocapillary hypercellularity and crescent formation. (**F**) Immunofluorescence microscopy showing intense mesangial IgA deposition using a FITC-conjugated anti-IgA antibody (×400).

Representative kidney biopsy images from patients included in the cohort are presented in Figure 1. The selected cases illustrate characteristic histopathological findings of pediatric IgA nephropathy, including mesangial hypercellularity, endocapillary proliferation, crescent formation, and mesangial IgA deposition on immunofluorescence.

Regarding therapeutic interventions, ACE inhibitors were administered to all patients during follow-up as part of nephroprotective management. They were used both for antiproteinuric purposes and as antihypertensive agents in patients with elevated blood pressure, independent of proteinuria status. Immunosuppressive treatment was administered to 78.6% of the cohort (*n* = 11), primarily consisting of oral corticosteroids. Following histological confirmation, MMF was introduced in selected cases, accompanied by gradual tapering of corticosteroids.

During follow-up, 10 patients discontinued corticosteroid therapy with stabilization of renal function, whereas 3 exhibited persistent severe proteinuria and remained on combination immunosuppressive therapy. One patient received additional cyclophosphamide pulses, with subsequent improvement in renal parameters. No clinical evidence of cyclophosphamide-related hemorrhagic cystitis or renal toxicity was documented during follow-up. Hypertension requiring additional antihypertensive treatment developed in one patient, while 7 patients continued ACE inhibitor therapy during follow-up. Overall, treatment strategies reflected individualized clinical decision-making based on disease severity, histopathological findings, and longitudinal response to therapy.

During a median follow-up period of approximately five years (range: 1–10 years), renal function remained largely stable in most patients (Table 3).

Stable renal function (<10% decline in eGFR) was observed in 42.9% of cases (*n* = 6). Mild decline (10–25%) was observed in 14.3% (*n* = 2), while progressive deterioration (>25% decline in eGFR) was identified in 21.4% (*n* = 3). Renal function improved in two patients (14.3%), whereas one patient (7.1%) experienced AKI requiring temporary dialysis with subsequent recovery of kidney function. Given the small sample size and descriptive study design, no conclusions regarding associations between histopathological findings and renal outcomes can be drawn.

Notably, improvement in renal function was observed in 14.3% of patients (*n* = 2), including one individual with initially reduced eGFR, indicating partial recovery of renal function during follow-up. In contrast, one patient (7.1%) developed severe AKI requiring temporary dialysis, followed by complete recovery of renal function, consistent with a reversible acute disease course.

**Figure 2:**
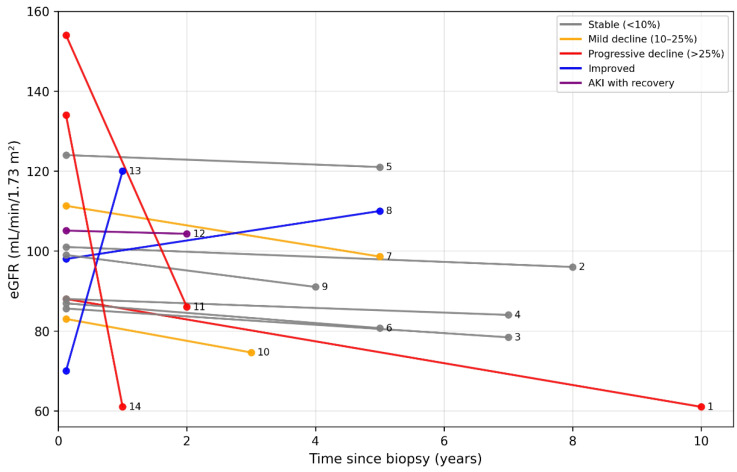
Longitudinal evolution of eGFR in pediatric IgA nephropathy. Each line represents the change in eGFR for an individual patient from baseline to last follow-up. eGFR was calculated using the bedside Schwartz equation.

Individual patient data demonstrated variability in renal function changes during follow-up, with most patients showing stable kidney function and a smaller number exhibiting decline or improvement (Figure 2).

Among the two patients with crescentic lesions (C1/C2), one experienced progressive decline in renal function during follow-up, whereas the other exhibited a mild decline. Given the very small number of patients with crescentic lesions, no conclusions regarding their prognostic significance can be drawn from the present cohort.

When stratified by follow-up duration, patients with longer follow-ups (>5 years) exhibited a slightly greater mean decline in eGFR compared to those with shorter follow-ups (−10.2% vs. −5.3%). However, the proportion of patients experiencing progressive decline was comparable between groups (20% vs. 22.2%), suggesting that follow-up duration alone did not substantially influence the observed outcomes. Overall, most patients in this cohort maintained stable kidney function during follow-up, although a clinically relevant minority experienced functional decline or acute severe presentations.

## 4. Discussion

Pediatric IgAN in our cohort was characterized by a largely stable renal course during mid-term follow-up, with most patients maintaining preserved or only minimally altered kidney function over time. However, this overall stability coexisted with clinically meaningful variability, as a subset of patients developed progressive renal impairment, while isolated but significant acute events, including dialysis-requiring AKI with subsequent full recovery, were also observed. Unlike large-scale multicenter studies that provide aggregate data, our study provides a descriptive patient-level assessment of renal function changes during follow-up. This approach allows visualization of individual patient courses that may not be apparent from summary measures alone.

In the present cohort, individual patients exhibited variable renal function courses, ranging from stability to decline or, in some cases, partial recovery. Within the limitations of this small retrospective cohort, these observations should be interpreted as descriptive and hypothesis-generating rather than prognostic. By integrating longitudinal clinical observations with biopsy-based characterization, the present study provides a patient-level description of disease evolution in a tertiary pediatric IgAN cohort. Although early outcomes were generally favorable, ongoing clinical follow-up remains important, particularly in patients with more severe clinical or histopathological features at presentation.

Our findings are broadly consistent with contemporary pediatric IgAN literature, which suggests that childhood-onset disease is typically characterized by a relatively favorable early course, albeit with substantial heterogeneity in long-term outcomes. In European cohorts, renal function is often preserved at diagnosis, and progression during childhood remains limited, although a proportion of patients may develop clinically relevant decline over extended follow-up [4]. More recent data further support a generally slow disease progression in children, with a minority of patients exhibiting adverse renal outcomes over mid-term observation periods [3]. In this context, the predominantly stable renal course observed in our cohort aligns with the overall pattern described in pediatric populations.

The proportion of patients demonstrating progressive eGFR decline in our study (21.4%) appears somewhat higher than that reported in larger series. This discrepancy likely reflects a significant referral-center bias. As a tertiary pediatric nephrology center, our cohort represents a high-acuity population with a high burden of active disease, as reflected by the high prevalence of E1 lesions (78.6%). In addition, variability in biopsy indications and treatment strategies across centers may contribute to differences in observed outcomes, as previously highlighted in pediatric IgAN studies [9]. Consistent with observations from routine clinical practice, variability in renal function changes was observed among individual patients during follow-up, highlighting the need for cautious interpretation and continued monitoring [10].

The relationship between histopathological findings and clinical outcomes in our cohort provides descriptive observations regarding the coexistence of Oxford MEST-C lesions and different clinical courses in pediatric IgAN. Mesangial hypercellularity (M1) was present in all patients and therefore could not serve as a discriminatory feature between different clinical outcomes observed during follow-up. Consequently, no conclusions regarding the prognostic significance of M1 can be drawn from the present dataset. The discussion of M1 is provided only to place our findings within the context of the existing literature, where its prognostic value remains under investigation, particularly in pediatric populations.

In contrast, endocapillary hypercellularity (E1) and segmental sclerosis (S1) were frequently observed and contributed to the overall histopathological characterization of the cohort. However, given the descriptive design of the study, the absence of formal statistical analyses, and the limited sample size, no inferences regarding potential associations between specific MEST-C lesions and renal outcomes can be made.

A substantial proportion of patients (71.4%) remained stable or improved despite the high histological burden, although the present study was not designed to assess treatment responsiveness or treatment effects. Because treatment strategies were individualized and most patients received some form of immunomodulatory and/or antiproteinuric therapy, the relative contribution of disease-related factors and treatment-related influences to the observed renal function cannot be determined. However, no conclusions regarding the impact of treatment on renal outcomes can be drawn from these data.

Notably, tubular atrophy and crescentic lesions were observed in a small number of patients within the cohort. These findings should be considered descriptive observations only. Given the limited number of such cases, no conclusions regarding their prognostic significance can be drawn [11,12,13]. Overall, the MEST-C classification provided a standardized framework for describing histopathological findings in our cohort; however, its prognostic significance could not be evaluated in the present study.

Acute presentations in our cohort further highlight the dynamic nature of pediatric IgAN. One patient developed severe AKI requiring temporary dialysis, followed by complete recovery of renal function, while additional cases demonstrated meaningful improvement in eGFR during follow-up. These patterns of partial or complete recovery suggest that pediatric IgAN may be associated with abrupt yet potentially reversible fluctuations in renal function. Such observations are consistent with current pediatric-focused recommendations emphasizing disease heterogeneity and variability in clinical course [1,14]. From a pathophysiological perspective, the observed changes in renal function may also relate to the resolution of acute inflammatory episodes or the normalization of initial hyperfiltration states, as illustrated by one patient with an exceptionally high baseline eGFR (154 mL/min/1.73 m^2^), in whom the subsequent decline may not necessarily represent true disease progression.

From a pathophysiological and clinical perspective, these observations may reflect early-stage disease at biopsy, where inflammatory processes predominate over irreversible structural damage. In addition, the variability observed in renal function during follow-up may reflect multiple factors, including differences in baseline disease severity, treatment exposure, and the resolution of acute inflammatory episodes [15]. The observed improvement in selected cases may also relate to normalization of initial hyperfiltration states, a phenomenon that requires cautious interpretation when assessing longitudinal eGFR changes. Overall, these findings align with emerging data supporting a multifactorial model of disease progression, integrating clinical, histological, and evolving biomarker-based risk stratification approaches [16,17]. Emerging computational approaches, including machine learning-based models integrating histological and clinical data, may further refine risk prediction in pediatric IgAN [18].

These observations carry important clinical implications. The predominance of stable renal function suggests that many patients maintained preserved kidney function during follow-up [1,14,19]. At the same time, the presence of a clinically relevant minority with progressive decline highlights the need for careful longitudinal monitoring. Given the descriptive nature of the study, no conclusions regarding risk stratification or individualized prognostic assessment can be drawn.

The strengths of this study lie in the meticulous use of a biopsy-confirmed pediatric cohort and the standardized application of the Oxford MEST-C classification, which provides a consistent and internationally recognized clinicopathological framework [6]. Beyond simple aggregate data, the availability of longitudinal follow-up allowed a descriptive patient-level characterization of renal function during follow-up. This approach complements summary data by illustrating the range of outcomes observed within the cohort. Furthermore, the findings provide descriptive information from routine clinical practice within a tertiary pediatric nephrology center [4,9].

Several limitations should be acknowledged, including the small sample size and single-center design, which may limit the generalizability of the findings. However, these characteristics enabled a detailed, case-by-case analysis of a high-acuity population. The retrospective nature of the study introduces potential selection and information bias. In addition, although the follow-up duration was adequate for mid-term assessment and extended up to a decade in some cases, it may not fully capture long-term progression into adulthood. It should also be acknowledged that patients with shorter follow-up may not yet have manifested clinically significant disease progression. Additionally, the inherent variability in follow-up intervals and therapeutic strategies, as commonly encountered in routine clinical practice, may have influenced individual outcomes, a challenge frequently noted in other pediatric IgAN cohorts [10]. Importantly, the heterogeneity of clinical presentations and treatment exposures limits the ability to distinguish disease-related effects from treatment-related influences on renal outcomes. Furthermore, the inclusion of patients with diverse clinical phenotypes, ranging from mild urinary abnormalities to nephrotic syndrome and AKI, may have contributed to the variability in renal outcomes observed during follow-up and should be considered when interpreting the findings. Similarly, the absence of an untreated comparison group precludes any assessment of the independent effects of therapeutic interventions on renal outcomes.

## 5. Conclusions

Taken together, this pediatric IgAN cohort followed a predominantly stable renal course over a median five-year follow-up, although a clinically significant subset of patients exhibited progressive decline or acute severe presentations. Within the limitations of this small retrospective, single-center cohort, variability in renal function evolution was observed among individual patients. These findings should be interpreted as descriptive and hypothesis-generating rather than prognostic. While the integration of longitudinal clinical observations and MEST-C histopathological features may provide useful clinical context, the present study was not designed to establish independent prognostic associations. Larger multicenter studies with standardized treatment protocols and longer follow-up are needed to better define determinants of long-term renal outcomes in pediatric IgAN.

## Figures and Tables

**Table 1:** Baseline clinicopathological characteristics of the IgAN cohort.

Oxford MEST-C	Hematuria at Presentation	Proteinuria at Presentation	Clinical Phenotype	Sex	Age, Years	Year of Biopsy	#
M1E1S1T0C0	Macroscopic	Nephrotic range	Steroid-resistant nephrotic syndrome	F	3.5	2016	1
M1E0S0T0C0	Macroscopic	Mild	Synpharyngitic hematuria	M	12	2018	2
M1E1S1T0C0	Macroscopic	Nephrotic range	Nephritic presentation with nephrotic-range proteinuria	F	15	2019	3
M1E1S1T0C0	Microscopic	Significant, non-nephrotic	Proliferative IgAN	F	13	2019	4
M1E0S0T0C0	Microscopic	Mild	Mild IgAN/autoimmune background	F	13	2021	5
M1E1S1T0C0	Macroscopic	Minimal/mild	Classic synpharyngitic IgAN	M	15	2021	6
M1E1S1T0C1	Macroscopic	Minimal/mild	Mild-to-active IgAN	M	7.5	2021	7
M1E0S0T0C0	Microscopic	Absent	IgAN with autoimmune thrombocytopenia	F	18	2021	8
M1E1S0T0C0	Microscopic	Nephrotic range	Steroid-dependent nephrotic syndrome/IgA overlap	M	4	2022	9
M1E1S1T0C0	Macroscopic	Nephrotic range	Infection-triggered IgAN	F	10	2023	10
M1E1S1T0C0	Macroscopic	Significant/episodic	Early active IgAN	M	13	2024	11
M1E1S0T0C0	Macroscopic	Moderate	Severe acute IgAN with AKI	M	10	2024	12
M1E1S0T0C0	Macroscopic	Moderate	Infection-associated IgAN	F	7.5	2025	13
M1E1S1T1C2	Macro- and microscopic	Nephrotic range	Advanced/progressive IgAN	M	14	2025	14

Nephrotic-range proteinuria was defined as >1000 mg/m^2^/day. Synpharyngitic hematuria refers to macroscopic hematuria occurring concurrently with upper respiratory tract infection. Baseline characteristics were recorded at the time of biopsy. Age refers to age at diagnosis (kidney biopsy). IgAN: immunoglobulin A nephropathy; AKI: acute kidney injury; MEST-C: Oxford classification [mesangial hypercellularity (M), endocapillary hypercellularity (E), segmental sclerosis (S), tubular atrophy/interstitial fibrosis (T), cellular/fibrocellular crescents (C)].

**Table 2:** Distribution of Oxford MEST-C lesions.

*n* (%) *	Lesion
14 (100%)	M1
11 (78.6%)	E1
8 (57.1%)	S1
1 (7.1%)	T1
2 (14.3%)	C1/C2
1 (7.1%)	C1
1 (7.1%)	C2

M: mesangial hypercellularity; E: endocapillary hypercellularity; S: segmental sclerosis; T: tubular atrophy/interstitial fibrosis; C: cellular or fibrocellular crescents. * Values are presented as number (percentage) of patients.

**Table 3:** Renal function evolution in pediatric IgAN.

Interpretation	ΔeGFR (%)	eGFR Follow-Up (mL/min/1.73 m^2^)	eGFR Baseline (mL/min/1.73 m^2^)	Follow-Up (Years)	#
Progressive decline	−30.7%	61	88	10	1
Stable	−5.0%	96	101	8	2
Stable	−8.4%	78.4	85.6	7	3
Stable	−4.5%	84	88	7	4
Stable	−2.4%	121	124	5	5
Stable	−7.1%	80.7	86.9	5	6
Mild decline	−11.4%	98.6	111.3	5	7
Improved	12.20%	110	98	5	8
Stable	−8.1%	91	99	4	9
Mild decline	−10.1%	74.6	83	3	10
Progressive decline ^1^	−44.2%	86	154	2	11
AKI requiring dialysis ^2^	−0.8%	104.3	105.1	2	12
Improved	71.40%	120	70	1	13
Progressive decline	−54.5%	61	134	1	14

^1^ The decline in eGFR should be interpreted with caution, as the baseline value (154 mL/min/1.73 m^2^) was suggestive of hyperfiltration, and the subsequent decrease may partly reflect normalization from an initially elevated eGFR rather than unequivocal disease progression. ^2^ The patient developed acute kidney injury requiring temporary dialysis, followed by full recovery of renal function. eGFR: estimated glomerular filtration rate (bedside Schwartz equation); ΔeGFR (%): percentage change from baseline; AKI: acute kidney injury. Renal outcomes were categorized as stable (<10% decline in eGFR), mild decline (1025%), and progressive decline (>25%).

## Data Availability

The data supporting the findings of this study are not publicly available due to privacy and ethical restrictions but are available from the corresponding author upon reasonable request.

## Abbreviations

The following abbreviations are used in this manuscript:

IgAN	Immunoglobulin A nephropathy
MEST-C	Mesangial hypercellularity, endocapillary hypercellularity, segmental glomerulosclerosis, tubular atrophy, and interstitial fibrosis-crescents
CKD	Chronic kidney disease
eGFR	Estimation of glomerular filtration rate
AKI	Acute kidney injury
MMF	Mycophenolate mofetil
ACE	Angiotensin-converting enzyme
